# Translation Initiation Regulated by RNA-Binding Protein in Mammals: The Modulation of Translation Initiation Complex by Trans-Acting Factors

**DOI:** 10.3390/cells10071711

**Published:** 2021-07-06

**Authors:** Akira Fukao, Takumi Tomohiro, Toshinobu Fujiwara

**Affiliations:** Department of Pharmacy, Kindai University, Kowakae 3-4-1, Higashi-Osaka City 577-8502, Japan; fukao@phar.kindai.ac.jp (A.F.); tomohiro@phar.kindai.ac.jp (T.T.)

**Keywords:** translation initiation, RNA-binding protein, microRNA, AU-rich element, poly(A)-binding protein, signaling pathway

## Abstract

Protein synthesis is tightly regulated at each step of translation. In particular, the formation of the basic cap-binding complex, eukaryotic initiation factor 4F (eIF4F) complex, on the 5′ cap structure of mRNA is positioned as the rate-limiting step, and various cis-elements on mRNA contribute to fine-tune spatiotemporal protein expression. The cis-element on mRNAs is recognized and bound to the trans-acting factors, which enable the regulation of the translation rate or mRNA stability. In this review, we focus on the molecular mechanism of how the assembly of the eIF4F complex is regulated on the cap structure of mRNAs. We also summarize the fine-tuned regulation of translation initiation by various trans-acting factors through cis-elements on mRNAs.

## 1. Introduction

**Figure 1:**
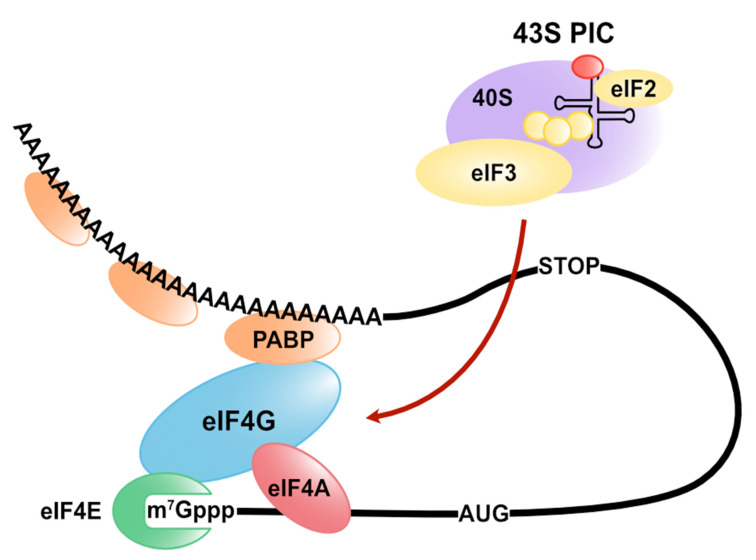
The formation of the basic translation initiation complex in eukaryotes. The eukaryotic translation initiation factor (eIF) 4F complex (which consists of eIF4E, eIF4G, and eIF4A) is assembled on the 5′ cap of mRNAs with PABP. The 43S PIC consists of 40S, a small ribosomal subunit, and several eIFs, such as eIF3 and eIF2.

Gene expression involves protein synthesis. The translation can be divided into four main events: initiation, elongation, termination, and ribosome recycling. Initiation begins with the binding of the ribosome to mRNA and the recognition of the start codon. In eukaryotes, the molecular mechanism of initiation is more complex than that of prokaryotes [1,2,3,4,5]. Eukaryotic mRNAs have a cap structure at the 5′ end and a poly(A) tail at the 3′ end. The cap-dependent, canonical translation is initiated when the eukaryotic translation initiation factor (eIF) 4F complex is assembled on the 5′ cap of mRNAs. The eIF4F complex consists of eIF4E (cap-binding), eIF4G (platform for eIFs), and eIF4A (RNA unwinding). eIF4G has binding sites of eIF4E, eIF4A, eIF3, and poly(A)-binding protein (PABP). The interaction between PABP and eIF4G results in the circularization of mRNA, enhances the binding affinity of the eIF4F complex for the 5′ cap, and facilitates the 43S pre-initiation complex (PIC) binding to the mRNA [6,7]. Since the formation of the eIF4F complex (including eIF4G/PABP interaction) is a rate-limiting step of cap-dependent translation initiation, it is a molecular target of translational control (Figure 1). Some trans-acting factors, such as RNA-binding proteins and microRNAs (miRNAs), regulate translation through the direct or indirect interaction with a basic cap-binding complex to modulate the translation initiation complex. This review summarizes the molecular mechanism of translation regulation via the stability control of the cap-binding complex by trans-acting factors and cis-elements on mRNA.

## 2. RNA-Binding Protein-Mediated Translation Regulation

RNA-binding proteins are necessary for the posttranscriptional regulation of gene expression through the control of mRNA stability and translation. Each RNA-binding protein binds to the specific cis-element on mRNAs [8,9,10]. AU-rich elements (AREs) are cis-elements that promote mRNA destabilization following their deadenylation induced by deadenylase complexes, such as carbon catabolite repression 4/negative on TATA-less (CCR4/NOT) and PAN2-PAN3 [11].

**Figure 2:**
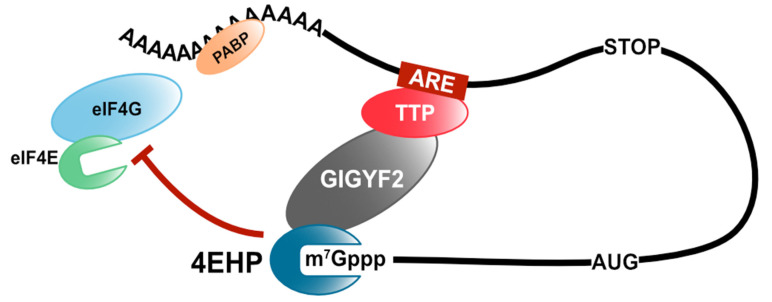
TTP-mediated translation repression. TTP recruits the GIGYF2/4EHP complex to the target mRNA and inhibits the assembly of eIF4F.

Tristetraprolin (TTP), also known as zinc finger binding protein 36 (ZFP36) of the TIS11 (TPA-induced sequence) family, is an ARE-binding protein (ARE-BP) that induces deadenylation of target mRNAs by the CCR4/NOT complex [12]. TTP has a tandem zinc finger RNA-binding domain and binds to the 3′ untranslated region (UTR) of target mRNAs, such as tumor necrosis factor-α (TNF-α) and granulocyte-macrophage colony-stimulating factor (GM-CSF) [13,14]. TTP mediates the recruitment of the CCR4/NOT complex to the target mRNA through direct interaction with its subunits, CNOT1 and CNOT9 [15,16]. CNOT1 is a scaffold protein and plays a pivotal role in the deadenylase activity of the CCR4/NOT complex. Additionally, TTP interacts with one of the cap-binding proteins, 4EHP (also known as eIF4E2), via the direct binding to the growth factor receptor-bound protein 10-interacting glycine–tyrosine–phenylalanine domain protein 2 (GIGYF2) and inhibits the translation of target mRNAs [17,18]. Unlike eIF4E, 4EHP does not bind to eIF4G and form the eIF4F complex [19]. Moreover, the overexpression of 4EHP enhances TTP-mediated translational repression, whereas the overexpression of a cap-binding mutant 4EHP disrupts TTP-mediated translational repression [18]. Thus, TTP inhibits target mRNA translation by recruiting 4EHP, thereby disrupting the assembly of the eIF4F complex (Figure 2).

ZFP36 ring finger protein-like 1 (ZFP36L1) is also an ARE-BP, which is one of the TIS11 family proteins as TTP [12]. It has been reported that ZFP36L1 interacts with subunits of the CCR4/NOT complex and induces target mRNA decay [20]. ZFP36L1 also has a conserved sequence for the CNOT1-interacting motif (CIM) of TTP [16]. Our recent study demonstrates that ZFP36L1 induces deadenylation of the target mRNA by utilizing the CCR4/NOT complex via the direct interaction between ZFP36L1 and CNOT1 [21]. It was also shown that ZFP36L1 represses translation in a deadenylation-independent manner. Moreover, it was discovered that the ZFP36L1-mediated translation repression requires its CNOT1-binding. Additionally, we showed that ZFP36L1 does not interact efficiently with 4EHP or GIGYF2, unlike TTP. This suggests that the recruitment of 4EHP or GIGYF2 to the target mRNA is dispensable for ZFP36L1-mediated translation repression, or ZFP36L1-mediated translation repression occurs in a cap-independent manner. To test this possibility, we utilized the encephalomyocarditis virus (EMCV) IRES, which does not require eIF4E for its translation. Notably, ZFP36L1 failed to repress the EMCV IRES-driven translation, suggesting that cap-dependent translation initiation is a primary target in ZFP36L1-mediated translation repression [21]. 

Importantly, some ARE-BPs play stabilizing roles, whereas others play destabilizing roles. Hu proteins are highly conserved RNA-binding proteins in vertebrates. In mammals, there are four Hu proteins (named HuR, HuB, HuC, and HuD), and they all positively regulate the stability of ARE-containing mRNAs in contrast to the TIS11 family [22]. HuR is ubiquitously expressed and binds to AREs located in the target mRNA 3′ UTR or intron sequences. HuR controls alternative splicing for specific genes (such as ZNF207, GANAB, DST, and PTBP2) or stabilization of ARE-containing mRNAs (encoded c-Fos and c-Jun) in the 3′ UTR or microRNA precursor processing (miR-7, encoded in the hnRNP K exon) [23,24]. Additionally, HuR upregulates the protein level of p53 by binding to the 3′ UTR of p53 mRNA in response to irradiation with the short-wavelength UV light [25].

Alternatively, HuB, HuC, and HuD are expressed in neurons and play a pivotal role in neuronal differentiation [26,27]. The neuronal Hu proteins induce neuronal differentiation through a neuron-specific RNA regulatory system, such as the spatiotemporal control of protein synthesis. Previous studies have shown that Hu proteins regulate the stability and translation of multiple target mRNAs, such as neurofilament M (NF-M), growth-associated protein-43 (GAP-43), tau, p21, and p27 [28,29,30]. GAP-43 mRNA is stabilized by HuD in a poly (A)-tail length-dependent manner. HuD can increase the half-life of GAP-43 mRNA containing ARE and a poly(A)-tail (at least 150 A nucleotides) in the 3′ UTR, but not short length poly(A)-tail (A30) [31]. Antic et al. reported that HuB binds to the 3′ UTR of NF-M mRNA, and NF-M mRNA is recruited efficiently to heavy polysomes in the presence of HuB [28]. Yano and colleagues revealed that HuB binds to the ARE in the 3′ UTR of p21 mRNA and increases p21 protein levels in mouse neuroblastoma N1E-115 cells. On the contrary, heterogeneous nuclear ribonucleoprotein K (hnRNP K) represses the translation of p21 mRNA by binding to the CU-rich region in the 3′ UTR. They also found that hnRNP K directly interacts with HuB. Interestingly, the binding sites of HuB and hnRNP K are adjacent in 3′ UTR of p21 mRNA, and they interact directly. They, therefore, proposed the model that hnRNP K antagonizes HuB mediated translation stimulation via their protein–protein interactions, although the underlying mechanism is unknown [30].

Our previous study showed that HuD enhances cap-dependent translation, and its activity of translation stimulation is necessary for neurite outgrowth in PC12 cells [32]. Hu proteins have three RNA-binding domains (RBDs, from the N-terminal RBD1 to the C-terminal RBD3) and a linker region between RBD2 and RBD3. It was reported that RBD1 and RBD2 bind to ARE, while RBD3 can interact with the poly(A)-tail and stabilize the mRNA–protein complex [33,34,35]. We revealed that HuD interacts with the cap-binding complex and that the HuD is co-sedimented with heavy polysomes, depending on its poly (A) binding ability [32]. The key is the interaction of HuD with actively translating poly(A) mRNAs but not with ribosomes. These observations prompted us to hypothesize that HuD might stimulate translation initiation of cap-poly(A) mRNA. We also found that HuD associates with the cap-binding complex via a direct binding to eIF4A. The eIF4A-binding activity of HuD is essential for the neurite outgrowth of PC12 cells and HuD-mediated translation activation. Thus, these findings showed that HuD stimulates the cap-poly(A) mRNA translation in a poly(A)- and eIF4A-binding-dependent manner [32]. In contrast, HuD represses the translation of p27 and preproinsulin (Ins2) mRNAs [36,37,38]. Although the molecular mechanism of translation repression by HuD is unclear, it was reported that an internal ribosome entry site (IRES) located in the 5′ UTR of p27 mRNA [36] and a short RNA sequence spanning positions 52–73 in the 5′ UTR of Ins2 mRNA [37,38] are required for HuD-binding. From this report, HuD can control the translation as a negative or positive regulator by forming a different complex on the target mRNA via the sequence and partner it binds to. IRES is the sequence that can initiate translation in a cap-independent manner and is also known to form various complexes via the binding to IRES-trans acting factors (ITAFs) [39].

**Figure 3:**
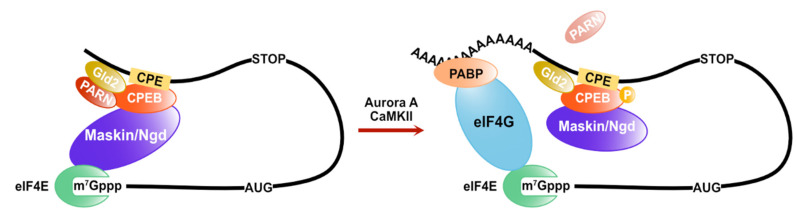
The regulation of the translation and the length of poly(A)-tail on CPE-containing mRNAs. CPEB proteins interact with maskin/Ngd and inhibit the assembly of eIF4F. CPEB protein is phosphorylated by Aurora A or CaMKII and induces the polyadenylation of CPE-containing mRNAs.

Cytoplasmic polyadenylation elements (CPEs) are also cis-elements in the 3′ UTR of mRNAs to control its translation rate. CPEs are recognized by CPEB (CPE binding) family proteins. Their functions are conserved from invertebrates to vertebrates [40,41]. The translational control mediated by CPEB proteins is required for early development and neuronal synaptic plasticity. In *Xenopus* oocytes, the CPEB proteins form a prominent complex on the 3′UTR of CPE-containing mRNAs with various factors in regulating poly (A) tail length and translation initiation. Maskin is a key protein, one of the CPEB protein interacting factors, and it represses translation via inhibition of the eIF4F complex assembly by directly binding to eIF4E [42]. Additionally, CPEB proteins interact with Gld2, a poly(A) polymerase (PAP) and poly(A)-specific ribonuclease (PARN) [43]. Although PARN is a cap-interacting protein and its binding stimulates the deadenylase activity [44,45,46], PARN interacts with maskin and eIF4E via the CPEB protein and competes with Gld2, leading to poly(A)-tail shortening of CPE-containing mRNAs [43]. When PARN dissociates from the CPEB complex via the phosphorylation of CPEB proteins, Gld2 elongates the poly(A)-tail of CPE-containing mRNAs. As a result, the amount of PABP increases, and eIF4E-interacting proteins on CPE-containing mRNAs replace maskin with eIF4G in a PABP-dependent manner [42]. CPEB proteins also regulate the translation of CPE-containing mRNAs via the poly(A)-tail length control through the competition of PARN and Gld2 in *Drosophila* and mammal neurons. However, the eIF4E-binding protein in the neural CPEB complex is neuroguidin (Ngd) rather than maskin [47]. Ngd is widely expressed in the nervous system and interacts with eIF4E and CPEB protein as maskin. Moreover, Ngd can repress translation in a CPE-dependent manner [48] (Figure 3).

## 3. microRNA-Mediated Translation Regulation

MicroRNAs are endogenous and small non-coding RNAs (20 bases) that bind to target mRNAs with a complementary sequence and regulate gene expression. Instead of functioning alone, miRNAs regulate gene expression by forming miRISC (miRNA-induced silencing complex) with Ago (Argonaute) proteins, and miRISC induces translation repression and destabilization of target mRNAs [49,50,51,52,53,54,55]. It has been shown that the GW182 protein is important for gene silencing by miRISC in animals. The GW182 protein interacts with the target mRNA by binding to the Ago protein and functions as a scaffold for PABP and two deadenylation complexes (CCR4/NOT and PAN2-PAN3) [56,57,58,59,60,61,62,63,64,65]. Some experiments with the knockdown of components or the dominant negative mutant overexpression of CCR4/NOT and PAN2-PAN3 complexes revealed that the CCR4/NOT complex is primarily involved in miRNA-mediated deadenylation [66,67,68]. After deadenylation, mRNA degradation in the 5′to 3′direction occurs, which requires the decapping of target mRNAs. MiRISC controls decapping by recruiting various factors involved in the decapping of target mRNAs. In mammals, DCP2, the catalytic subunit of decapping, DCP1, DDX6, and EDC4, which are involved in the activation of decapping, interacts with the Ago proteins [69].

Many studies with different organisms and methods have shown that gene silencing by miRNAs induces mRNA degradation and a translation repression pathway. GW182 suppresses translation even for mRNAs without a poly (A)-tail in a CCR4/NOT complex-dependent manner [61,62]. This result indicates that GW182 plays an essential role in miRNA-mediated translation repression. Additionally, since CAF1, which is a deadenylase of the CCR4/NOT complex, mediates the deadenylation-independent translation repression, the CCR4/NOT complex is also vital for miRNA-mediated translation repression [70,71]. Several groups have shown that CNOT1 binds explicitly to the DEAD-box RNA helicase DDX6, also known as a decapping activator mentioned above. DDX6 represses the translation at the initiation step and elongation step [72,73]. In addition, DDX6 recruits the eIF4E-binding protein 4E-T to the target mRNA, and 4E-T represses the translation via its eIF4E-binding domain [74,75].

**Figure 4:**
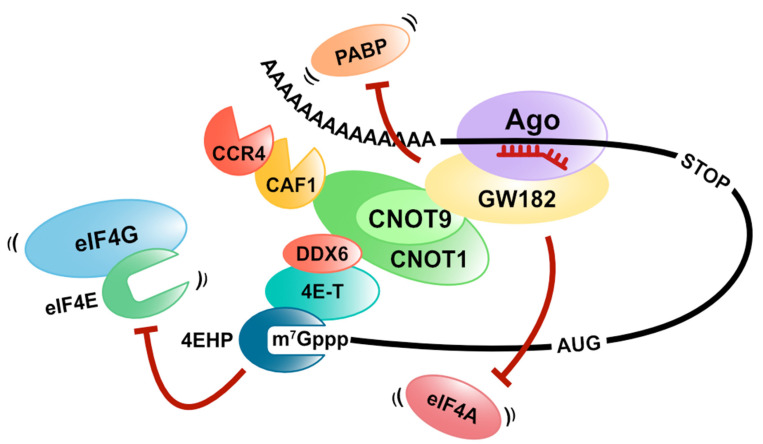
MiRNA-mediated translation repression. MiRISC (including GW182) represses the translation in a deadenylation-independent manner via the dissociation of PABP, eIF4As, or eIF4E.

Furthermore, Chapat et al. and Chen et al. reveal that the cap-binding activity of 4EHP contributes to the miRNA-mediated translation repression through the CCR4/NOT complex [76,77]. They also show that 4EHP competes with eIF4E for the 4E-T binding, while the cap-binding affinity of 4EHP increased by 4E-T binding [76]. These reports suggest that miRNA-mediated translation repression is caused by crippling eIF4F on target mRNAs by recruiting DDX6, 4E-T, and 4EHP with CNOT1 as a scaffold in a GW182-dependent manner (Figure 4).

**Figure 5:**
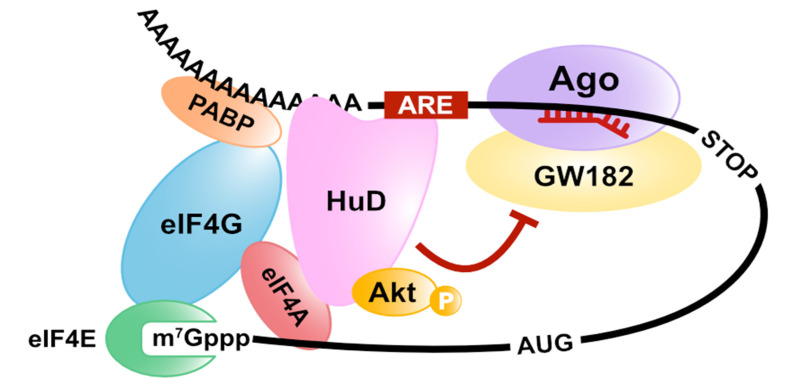
HuD-mediated translation stimulation and miRNA inhibition. HuD interacts with the basic cap-binding complex via the binding to eIF4A and poly(A)-tail and inhibits miRNA-mediated translation repression through the eIF4A-binding.

Alternatively, we have demonstrated another model of miRNA-mediated translation repression with a different approach [78]. First, we isolated an mRNA-protein complex, including miRISC on target mRNAs, by an in vitro translation system arranged with GRNA affinity chromatography (mRNA pull-down using the specific interaction between lambda N peptides and BoxB sequences [79]) and analyzed its constituent factors. We discovered that both eIF4AI and eIF4AII were dissociated from the translation initiation complex on the miRISC-bound mRNA [78]. Furthermore, the excess amount of eIF4AI and eIF4AII attenuates miRNA-mediated translation repression. These results suggest that miRISC dissociates eIF4AI and eIF4AII from the eIF4F complex or inhibits eIF4F formation on target mRNAs (Figure 4). To test these possibilities, we used a pharmacological eIF4A inhibitor, silvestrol, which immobilizes eIF4A onto mRNA [80,81,82]. We observed that eIF4As still remain associated with mRNAs even in the presence of both miRNA and silvestrol. This result strongly suggests that miRNA displaces eIF4As from mRNA rather than inhibiting initial recruitment of eIF4A to mRNAs. We next used the feature of HuD as a tool to prove that the dissociation of eIF4AI and eIF4AII from the eIF4F complex is essential for miRNA-mediated translation repression [78]. Binding to eIF4A is necessary for HuD-mediated translation stimulation [32]. Therefore, HuD was expected to act antagonistically with the miRNA mechanism that also targets eIF4A. As expected, we confirmed that HuD inhibits the miRNA-mediated translation repression and the dissociation of eIF4AI and eIF4AII from the eIF4F complex in an eIF4A-binding dependent manner (Figure 5). In summary, we conclude that the translational inhibitory effect of miRNAs in humans is exerted by dissociating eIF4AI and eIF4AII from the target mRNA [78]. Fukaya and Tomari also demonstrated that fly Ago1-RISC induces the dissociation of eIF4A from the eIF4F complex in *Drosophila*, using a different approach (site-specific UV crosslinking analysis) [83]. These reports suggest that eIF4A is targeted by miRNA-mediated translation repression, and this model is conserved in animals.

## 4. Translation Regulation via PABP-Binding

Thus far, the molecular mechanism directly involved in the eIF4F formation for translational regulation by RNA-binding proteins and miRNAs has been described. However, the presence of PABP on mRNAs is indispensable for the efficiency of eIF4F formation, and there are many studies on translation repression on specific mRNAs by inhibiting the function of PABP. For example, PABP interacting protein 2 (Paip2), a negative translational regulator, directly binds to PABP via PABP interacting motif 1 (PAM1) and PAM2. It was reported that Paip2 represses translation through the release of PABP from the poly(A) tail of mRNA [84,85]. Msi, the *Drosophila* homolog of musashi, is an RNA-binding protein containing two RNA recognition motifs. In mammals, there are two members of the Msi family proteins, Msi1 and Msi2. Each Msi protein has a different expression pattern that Msi1 expresses in neuronal stem cells, and Msi2 expresses ubiquitously [86,87,88]. Kawahara et al. showed that Msi1 inhibits the translation of *m-Numb* mRNA via its PABP-binding ability. Msi1 binds to PABP directly at the C-terminal region of Msi1 and competes with eIF4G [89]. This implies that Msi1 dissociates PABP from the translation initiation complex and destabilizes the eIF4F complex on target mRNAs. In contrast, Cragle and MacNicol reported that Msi1 is an activator of translation in progesterone-stimulated *Xenopus* oocytes [90]. They also showed that Msi1 interacts with Gld2 and that Msi1 stimulates translation via the polyadenylation of the target mRNA, such as CPEB proteins.

As previously described, GW182 is an essential protein for miRNA-mediated translation repression and deadenylation in animals. GW182 directly interacts with PABP through PAM2 located in the C-terminal silencing domain and competes with eIF4G to bind with PABP in *Drosophila* [56,58,59]. Although deadenylation induces the dissociation of PABP from target mRNAs, it has been reported that GW182 displaces PABP from the target mRNA in the absence of deadenylation [57,91] (Figure 4). In addition, disassembly of PABP from poly(A)-tail requires interaction between GW182 and the CCR4/NOT complex. Moreover, Yi et al. suggest that PABP concerts with the CCR4/NOT complex to regulate mRNA deadenylation. CAF1 and CCR4 are catalytic subunits of the CCR4/NOT complex, and CCR4 requires CAF1 for the interaction with CNOT1. They showed that CAF1 and CCR4 have a distinct activity for deadenylation using the CCR4/NOT complex, including each catalytic mutant purified from mammalian cells. CAF1 untrims the PABP-bound poly(A) tail. Alternatively, the deadenylase activity of CCR4 increases in the presence of PABP [92]. These results suggest that the miRNA-mediated dissociation of PABP and deadenylation on target mRNAs depends on the usage of CCR4 or CAF1 within the CCR4/NOT complex.

## 5. Translation Regulation via Phosphorylation of RNA-Binding Proteins

In eukaryotes, the assembly of the eIF4F complex is regulated through the phosphorylation of various eIFs. 4E-BPs are eIF4E-binding proteins that inhibit the formation of the eIF4F complex via competition with eIF4G [93]. The mechanistic/mammalian target of rapamycin (mTOR) directly phosphorylates and inactivates 4E-BPs, followed by increasing the eIF4F complex [94,95]. Programmed cell death 4 (PDCD4) binds to eIF4A and inhibits translation by reducing eIF4A available to the eIF4F complex [96]. PDCD4 is phosphorylated by the mTOR pathway and inhibits the binding to eIF4A [97,98]. These are examples of phosphorylation-mediated regulatory mechanisms of the eIF4F complex formation for general mRNAs. In higher eukaryotes, the regulation of spatiotemporal protein synthesis requires the cooperative action of the signaling pathway and RNA-binding proteins.

In *Xenopus* oocytes, progesterone stimulates a serine/threonine kinase known as Aurora A. It phosphorylates CPEB at serine 174, and then phospho-CPEB induces polyadenylation of CPE-containing mRNAs via Gld2 [99,100]. Moreover, in the brain, the CPEB complex on CPE-containing mRNAs, including Ngd but not maskin, regulates synaptic plasticity and hippocampal-dependent memories. Ca^2+^/calmodulin-dependent protein kinase II (CaMKII) phosphorylates CPEB via activation of the N-methyl-D-aspartate receptor (NMDAR) [101,102]. The phosphorylation of the CPEB protein induces polyadenylation and translation of CPE-containing mRNAs by a similar mechanism as a CPEB complex, including maskin [48]. Protein kinase B (PKB/Akt) phosphorylates ZFP36L1 and inactivates its ability of mRNA decay [103,104]. It was reported that the phosphorylation of ZFP36L1 increased the interaction with 14-3-3 proteins, which are multifunctional adaptor proteins. Additionally, the ZFP36L1-mediated translation repression and deadenylation require interaction with CNOT1 [21]. These results imply that 14-3-3 proteins inhibit the recruitment of the CCR4/NOT complex to target mRNAs via the phosphorylation of ZFP36L1. Protein kinase C (PKC) is a serine/threonine kinase, which is ubiquitously expressed and phosphorylates HuD [105,106]. The phosphorylation of HuD induced stabilizes the target mRNA (GAP-43 mRNA) and increases the GAP-43 protein level [105]. It has been shown that HuD upregulates cap- and poly(A)-dependent translation via direct interaction with eIF4A (see Section 2). We previously showed that the PI3K/Akt pathway is involved in the ability of HuD to induce differentiation of PC12 cells by overexpressing a dominant negative mutant of Akt1. HuD directly and specifically interacts with the active (phosphorylated) form of Akt1, and this interaction is essential for HuD-mediated differentiation of PC12 cells [107]. Notably, only neuronal Hu proteins (HuB, HuC, and HuD) can bind to the active Akt1 but not to ubiquitous HuR. Furthermore, it was discovered that the Akt1-binding region in HuD is a linker region between RBD2 and RBD3, which includes neuronal-specific sequences, using chimeric mutants by swapping the linker region between HuD and HuR. Moreover, we indicated that Akt1 does not phosphorylate HuD, although HuD specifically binds to the active Akt1 [107]. This suggests that HuD functions as a platform of signaling pathways onto specific mRNAs. The PI3K/Akt pathway activates mTOR, resulting in increased assembly of the eIF4F complex via the phosphorylation of 4E-BP and PDCD4 [94,95,96,97,98]. Additionally, PKB/Akt directly phosphorylates eIF4B [108], and mTOR activated by PKB/Akt also phosphorylates eIF4B (mediated by p70S6K) [109,110]. eIF4B is the accessory protein of eIF4A and can stimulate the helicase activity of eIF4A through its phosphorylation [111]. These reports may imply that HuD enhances the cap- and poly(A)-dependent translation by activating eIF4A helicase activity via the recruitment of active Akt1 to the eIF4F complex. Alternatively, eIF4B is directly phosphorylated by PKB/Akt at Ser422 and indirectly phosphorylated by the mTOR pathway at Ser406 [108]. In the brain, BC1 RNA, a brain cytoplasmic (BC) RNA that is a non-coding small RNA, regulates translation in cooperation with eIF4B [112]. BC1 RNA associates with eIF4B in the state of phosphorylation at Ser406 and inhibits translation. When eIF4B is dephosphorylated by protein phosphatase 2A (PP2A) at Ser406, BC1 RNA is released from eIF4B and begins translation [112]. This indicates that Ser406 of eIF4B is an important residue for the translation regulation in neurons. It is still unclear how the HuD-active Akt1 complex regulates translation, but the functional changes in the phosphorylated state of eIF4B in neurons are interesting.

## 6. Conclusions

Expression of genetic information that is transcribed from DNA into mRNA is controlled by complicated and sophisticated regulatory systems with various RNA-binding trans-acting factors (e.g., RNA-binding proteins and microRNAs) and determines higher order vital cellular phenomena in eukaryotes. Among these post-transcriptional gene expression regulations, fine-tuning of spatiotemporal protein synthesis is particularly essential for cell growth, development, and differentiation. To accomplish spatiotemporal translation, the regulation of the translation initiation step is very efficient because this step is the rate-limiting step of protein synthesis. There emerged evidence that RNA-binding proteins and microRNAs control the differentiation process of various cells via the regulation of spatiotemporal translation as a regulator of translation initiation. By having various RNA-binding motifs, RNA-binding proteins bind to a specific mRNA in a specific manner. In addition, hundreds of transcripts are microRNA targets, and many of them are regulated by multiple miRNAs. Therefore, to elucidate the molecular mechanism of spatiotemporal regulation of translation initiation, it is imperative to identify the primary sequence or structure of target mRNAs recognized by RNA-binding proteins and miRNAs. Recently developed methods, such as HITS-CLIP [113] and PAR-CLIP [114], to analyze direct interactions between RNA-binding proteins and RNAs in living cells enabled the comprehensive analysis of endogenous targets of RNA-binding proteins and microRNAs. In addition, ribosome profiling, a method based on deep-sequencing of mRNA fragments occupied by the 80S ribosome, has been established [115,116]. This method enabled comprehensive and quantitatively identification of the sequences covered by the 80S ribosome, and the translation level of mRNAs in living cells can be determined. Furthermore, recent revolutionary advances in cryo-electron microscopy will reveal the complex structure of the ribosome and the various ribosome binding factors at high resolution. These emerging innovative technologies will enable us to understand the entire network of interactions for cell-specific regulation of translation initiation in the near future.

## References

[1] Jackson R.J., Hellen C.U., Pestova T.V. (2010). The mechanism of eukaryotic translation initiation and principles of its regulation. Nat. Rev. Mol. Cell. Biol..

[2] Dever T.E., Green R. (2012). The elongation, termination, and recycling phases of translation in eukaryotes. Cold Spring Harb. Perspect. Biol..

[3] Hinnebusch A.G., Lorsch J.R. (2012). The mechanism of eukaryotic translation initiation: New insights and challenges. Cold Spring Harb. Perspect. Biol..

[4] Hinnebusch A.G. (2014). The scanning mechanism of eukaryotic translation initiation. Annu. Rev. Biochem..

[5] Shirokikh N.E., Preiss T. (2018). Translation initiation by cap-dependent ribosome recruitment: Recent insights and open questions. Wiley Interdiscip. Rev. RNA.

[6] Wells S.E., Hillner P.E., Vale R.D., Sachs A.B. (1998). Circularization of mRNA by eukaryotic translation initiation factors. Mol. Cell.

[7] Kahvejian A., Svitkin Y.V., Sukarieh R., M’Boutchou M.N., Sonenberg N. (2005). Mammalian poly(A)-binding protein is a eukaryotic translation initiation factor, which acts via multiple mechanisms. Genes Dev..

[8] Garneau N.L., Wilusz J., Wilusz C.J. (2007). The highways and byways of mRNA decay. Nat. Rev. Mol. Cell. Biol..

[9] Matoulkova E., Michalova E., Vojtesek B., Hrstka R. (2012). The role of the 3′ untranslated region in post-transcriptional regulation of protein expression in mammalian cells. RNA Biol..

[10] Mitchell S.F., Parker R. (2014). Principles and properties of eukaryotic mRNPs. Mol. Cell.

[11] Otsuka H., Fukao A., Funakami Y., Duncan K.E., Fujiwara T. (2019). Emerging Evidence of Translational Control by AU-Rich Element-Binding Proteins. Front. Genet..

[12] Lykke-Andersen J., Wagner E. (2005). Recruitment and activation of mRNA decay enzymes by two ARE-mediated decay activation domains in the proteins TTP and BRF-1. Genes Dev..

[13] Lai W.S., Blackshear P.J. (2001). Interactions of CCCH zinc finger proteins with mRNA: Tristetraprolin-mediated AU-rich element-dependent mRNA degradation can occur in the absence of a poly(A) tail. J. Biol. Chem..

[14] Lai W.S., Carballo E., Strum J.R., Kennington E.A., Phillips R.S., Blackshear P.J. (1999). Evidence that tristetraprolin binds to AU-rich elements and promotes the deadenylation and destabilization of tumor necrosis factor alpha mRNA. Mol. Cell. Biol..

[15] Bulbrook D., Brazier H., Mahajan P., Kliszczak M., Fedorov O., Marchese F.P., Aubareda A., Chalk R., Picaud S., Strain-Damerell C. (2018). Tryptophan-Mediated Interactions between Tristetraprolin and the CNOT9 Subunit Are Required for CCR4-NOT Deadenylase Complex Recruitment. J. Mol. Biol..

[16] Fabian M.R., Frank F., Rouya C., Siddiqui N., Lai W.S., Karetnikov A., Blackshear P.J., Nagar B., Sonenberg N. (2013). Structural basis for the recruitment of the human CCR4-NOT deadenylase complex by tristetraprolin. Nat. Struct. Mol. Biol..

[17] Fu R., Olsen M.T., Webb K., Bennett E.J., Lykke-Andersen J. (2016). Recruitment of the 4EHP-GYF2 cap-binding complex to tetraproline motifs of tristetraprolin promotes repression and degradation of mRNAs with AU-rich elements. RNA.

[18] Tao X., Gao G. (2015). Tristetraprolin Recruits Eukaryotic Initiation Factor 4E2 To Repress Translation of AU-Rich Element-Containing mRNAs. Mol. Cell. Biol..

[19] Morita M., Ler L.W., Fabian M.R., Siddiqui N., Mullin M., Henderson V.C., Alain T., Fonseca B.D., Karashchuk G., Bennett C.F. (2012). A novel 4EHP-GIGYF2 translational repressor complex is essential for mammalian development. Mol. Cell. Biol..

[20] Takahashi A., Adachi S., Morita M., Tokumasu M., Natsume T., Suzuki T., Yamamoto T. (2015). Post-transcriptional Stabilization of Ucp1 mRNA Protects Mice from Diet-Induced Obesity. Cell Rep..

[21] Otsuka H., Fukao A., Tomohiro T., Adachi S., Suzuki T., Takahashi A., Funakami Y., Natsume T., Yamamoto T., Duncan K.E. (2020). ARE-binding protein ZFP36L1 interacts with CNOT1 to directly repress translation via a deadenylation-independent mechanism. Biochimie.

[22] Hinman M.N., Lou H. (2008). Diverse molecular functions of Hu proteins. Cell. Mol. Life Sci..

[23] Lebedeva S., Jens M., Theil K., Schwanhäusser B., Selbach M., Landthaler M., Rajewsky N. (2011). Transcriptome-wide analysis of regulatory interactions of the RNA-binding protein HuR. Mol. Cell.

[24] Peng S.S., Chen C.Y., Xu N., Shyu A.B. (1998). RNA stabilization by the AU-rich element binding protein, HuR, an ELAV protein. EMBO J..

[25] Mazan-Mamczarz K., Galbán S., López de Silanes I., Martindale J.L., Atasoy U., Keene J.D., Gorospe M. (2003). RNA-binding protein HuR enhances p53 translation in response to ultraviolet light irradiation. Proc. Natl. Acad. Sci. USA.

[26] Akamatsu W., Okano H.J., Osumi N., Inoue T., Nakamura S., Sakakibara S., Miura M., Matsuo N., Darnell R.B., Okano H. (1999). Mammalian ELAV-like neuronal RNA-binding proteins HuB and HuC promote neuronal development in both the central and the peripheral nervous systems. Proc. Natl. Acad. Sci. USA.

[27] Akamatsu W., Fujihara H., Mitsuhashi T., Yano M., Shibata S., Hayakawa Y., Okano H.J., Sakakibara S., Takano H., Takano T. (2005). The RNA-binding protein HuD regulates neuronal cell identity and maturation. Proc. Natl. Acad. Sci. USA.

[28] Antic D., Lu N., Keene J.D. (1999). ELAV tumor antigen, Hel-N1, increases translation of neurofilament M mRNA and induces formation of neurites in human teratocarcinoma cells. Genes Dev..

[29] Aranda-Abreu G.E., Behar L., Chung S., Furneaux H., Ginzburg I. (1999). Embryonic lethal abnormal vision-like RNA-binding proteins regulate neurite outgrowth and tau expression in PC12 cells. J. Neurosci..

[30] Yano M., Okano H.J., Okano H. (2005). Involvement of Hu and heterogeneous nuclear ribonucleoprotein K in neuronal differentiation through p21 mRNA post-transcriptional regulation. J. Biol. Chem..

[31] Beckel-Mitchener A.C., Miera A., Keller R., Perrone-Bizzozero N.I. (2002). Poly(A) tail length-dependent stabilization of GAP-43 mRNA by the RNA-binding protein HuD. J. Biol. Chem..

[32] Fukao A., Sasano Y., Imataka H., Inoue K., Sakamoto H., Sonenberg N., Thoma C., Fujiwara T. (2009). The ELAV protein HuD stimulates cap-dependent translation in a Poly(A)- and eIF4A-dependent manner. Mol. Cell.

[33] Abe R., Sakashita E., Yamamoto K., Sakamoto H. (1996). Two different RNA binding activities for the AU-rich element and the poly(A) sequence of the mouse neuronal protein mHuC. Nucleic Acids Res..

[34] Chung S., Jiang L., Cheng S., Furneaux H. (1996). Purification and properties of HuD, a neuronal RNA-binding protein. J. Biol. Chem..

[35] Ma W.J., Chung S., Furneaux H. (1997). The Elav-like proteins bind to AU-rich elements and to the poly(A) tail of mRNA. Nucleic Acids Res..

[36] Kullmann M., Gopfert U., Siewe B., Hengst L. (2002). ELAV/Hu proteins inhibit p27 translation via an IRES element in the p27 5′UTR. Genes Dev..

[37] Lee E.K., Kim W., Tominaga K., Martindale J.L., Yang X., Subaran S.S., Carlson O.D., Mercken E.M., Kulkarni R.N., Akamatsu W. (2012). RNA-binding protein HuD controls insulin translation. Mol. Cell.

[38] Lee E.K., Kim W., Tominaga K., Martindale J.L., Yang X., Subaran S.S., Carlson O.D., Mercken E.M., Kulkarni R.N., Akamatsu W. (2020). RNA-Binding Protein HuD Controls Insulin Translation. Mol. Cell.

[39] Lloyd R.E. (2015). Nuclear proteins hijacked by mammalian cytoplasmic plus strand RNA viruses. Virology.

[40] Charlesworth A., Meijer H.A., de Moor C.H. (2013). Specificity factors in cytoplasmic polyadenylation. Wiley Interdiscip. Rev. RNA.

[41] Kozlov E., Shidlovskii Y.V., Gilmutdinov R., Schedl P., Zhukova M. (2021). The role of CPEB family proteins in the nervous system function in the norm and pathology. Cell Biosci..

[42] Cao Q., Richter J.D. (2002). Dissolution of the maskin-eIF4E complex by cytoplasmic polyadenylation and poly(A)-binding protein controls cyclin B1 mRNA translation and oocyte maturation. EMBO J..

[43] Kim J.H., Richter J.D. (2006). Opposing polymerase-deadenylase activities regulate cytoplasmic polyadenylation. Mol. Cell.

[44] Martînez J., Ren Y.G., Nilsson P., Ehrenberg M., Virtanen A. (2001). The mRNA cap structure stimulates rate of poly(A) removal and amplifies processivity of degradation. J. Biol. Chem..

[45] Wu M., Reuter M., Lilie H., Liu Y., Wahle E., Song H. (2005). Structural insight into poly(A) binding and catalytic mechanism of human PARN. EMBO J..

[46] Wu M., Nilsson P., Henriksson N., Niedzwiecka A., Lim M.K., Cheng Z., Kokkoris K., Virtanen A., Song H. (2009). Structural basis of m(7)GpppG binding to poly(A)-specific ribonuclease. Structure.

[47] Jung M.Y., Lorenz L., Richter J.D. (2006). Translational control by neuroguidin, a eukaryotic initiation factor 4E and CPEB binding protein. Mol. Cell. Biol..

[48] Udagawa T., Swanger S.A., Takeuchi K., Kim J.H., Nalavadi V., Shin J., Lorenz L.J., Zukin R.S., Bassell G.J., Richter J.D. (2012). Bidirectional control of mRNA translation and synaptic plasticity by the cytoplasmic polyadenylation complex. Mol. Cell.

[49] Bazzini A.A., Lee M.T., Giraldez A.J. (2012). Ribosome profiling shows that miR-430 reduces translation before causing mRNA decay in zebrafish. Science.

[50] Djuranovic S., Nahvi A., Green R. (2012). miRNA-mediated gene silencing by translational repression followed by mRNA deadenylation and decay. Science.

[51] Guo H., Ingolia N.T., Weissman J.S., Bartel D.P. (2010). Mammalian microRNAs predominantly act to decrease target mRNA levels. Nature.

[52] Baek D., Villén J., Shin C., Camargo F.D., Gygi S.P., Bartel D.P. (2008). The impact of microRNAs on protein output. Nature.

[53] Eulalio A., Huntzinger E., Izaurralde E. (2008). Getting to the root of miRNA-mediated gene silencing. Cell.

[54] Fabian M.R., Sonenberg N. (2012). The mechanics of miRNA-mediated gene silencing: A look under the hood of miRISC. Nat. Struct. Mol. Biol..

[55] Iwakawa H.O., Tomari Y. (2015). The Functions of MicroRNAs: mRNA Decay and Translational Repression. Trends Cell Biol..

[56] Fabian M.R., Mathonnet G., Sundermeier T., Mathys H., Zipprich J.T., Svitkin Y.V., Rivas F., Jinek M., Wohlschlegel J., Doudna J.A. (2009). Mammalian miRNA RISC recruits CAF1 and PABP to affect PABP-dependent deadenylation. Mol. Cell.

[57] Zekri L., Huntzinger E., Heimstädt S., Izaurralde E. (2009). The silencing domain of GW182 interacts with PABPC1 to promote translational repression and degradation of microRNA targets and is required for target release. Mol. Cell. Biol..

[58] Huntzinger E., Braun J.E., Heimstadt S., Zekri L., Izaurralde E. (2010). Two PABPC1-binding sites in GW182 proteins promote miRNA-mediated gene silencing. EMBO J..

[59] Jinek M., Fabian M.R., Coyle S.M., Sonenberg N., Doudna J.A. (2010). Structural insights into the human GW182-PABC interaction in microRNA-mediated deadenylation. Nat. Struct. Mol. Biol..

[60] Fabian M.R., Cieplak M.K., Frank F., Morita M., Green J., Srikumar T., Nagar B., Yamamoto T., Raught B., Duchaine T.F. (2011). miRNA-mediated deadenylation is orchestrated by GW182 through two conserved motifs that interact with CCR4-NOT. Nat. Struct. Mol. Biol..

[61] Braun J.E., Huntzinger E., Fauser M., Izaurralde E. (2011). GW182 proteins directly recruit cytoplasmic deadenylase complexes to miRNA targets. Mol. Cell.

[62] Chekulaeva M., Mathys H., Zipprich J.T., Attig J., Colic M., Parker R., Filipowicz W. (2011). miRNA repression involves GW182-mediated recruitment of CCR4-NOT through conserved W-containing motifs. Nat. Struct. Mol. Biol..

[63] Kuzuoglu-Ozturk D., Huntzinger E., Schmidt S., Izaurralde E. (2012). The Caenorhabditis elegans GW182 protein AIN-1 interacts with PAB-1 and subunits of the PAN2-PAN3 and CCR4-NOT deadenylase complexes. Nucleic Acids Res..

[64] Huntzinger E., Kuzuoglu-Ozturk D., Braun J.E., Eulalio A., Wohlbold L., Izaurralde E. (2013). The interactions of GW182 proteins with PABP and deadenylases are required for both translational repression and degradation of miRNA targets. Nucleic Acids Res..

[65] Christie M., Boland A., Huntzinger E., Weichenrieder O., Izaurralde E. (2013). Structure of the PAN3 pseudokinase reveals the basis for interactions with the PAN2 deadenylase and the GW182 proteins. Mol. Cell.

[66] Piao X., Zhang X., Wu L., Belasco J.G. (2010). CCR4-NOT deadenylates mRNA associated with RNA-induced silencing complexes in human cells. Mol. Cell. Biol..

[67] Eulalio A., Huntzinger E., Nishihara T., Rehwinkel J., Fauser M., Izaurralde E. (2009). Deadenylation is a widespread effect of miRNA regulation. RNA.

[68] Chen C.Y., Zheng D., Xia Z., Shyu A.B. (2009). Ago-TNRC6 triggers microRNA-mediated decay by promoting two deadenylation steps. Nat. Struct. Mol. Biol..

[69] Jonas S., Izaurralde E. (2013). The role of disordered protein regions in the assembly of decapping complexes and RNP granules. Genes Dev..

[70] Cooke A., Prigge A., Wickens M. (2010). Translational repression by deadenylases. J. Biol. Chem..

[71] Bawankar P., Loh B., Wohlbold L., Schmidt S., Izaurralde E. (2013). NOT10 and C2orf29/NOT11 form a conserved module of the CCR4-NOT complex that docks onto the NOT1 N-terminal domain. RNA Biol..

[72] Mathys H., Basquin J., Ozgur S., Czarnocki-Cieciura M., Bonneau F., Aartse A., Dziembowski A., Nowotny M., Conti E., Filipowicz W. (2014). Structural and biochemical insights to the role of the CCR4-NOT complex and DDX6 ATPase in microRNA repression. Mol. Cell.

[73] Chen Y., Boland A., Kuzuoglu-Ozturk D., Bawankar P., Loh B., Chang C.T., Weichenrieder O., Izaurralde E. (2014). A DDX6-CNOT1 complex and W-binding pockets in CNOT9 reveal direct links between miRNA target recognition and silencing. Mol. Cell.

[74] Kamenska A., Lu W.T., Kubacka D., Broomhead H., Minshall N., Bushell M., Standart N. (2014). Human 4E-T represses translation of bound mRNAs and enhances microRNA-mediated silencing. Nucleic Acids Res..

[75] Kamenska A., Simpson C., Vindry C., Broomhead H., Bénard M., Ernoult-Lange M., Lee B.P., Harries L.W., Weil D., Standart N. (2016). The DDX6-4E-T interaction mediates translational repression and P-body assembly. Nucleic Acids Res..

[76] Chapat C., Jafarnejad S.M., Matta-Camacho E., Hesketh G.G., Gelbart I.A., Attig J., Gkogkas C.G., Alain T., Stern-Ginossar N., Fabian M.R. (2017). Cap-binding protein 4EHP effects translation silencing by microRNAs. Proc. Natl. Acad. Sci. USA.

[77] Chen S., Gao G. (2017). MicroRNAs recruit eIF4E2 to repress translation of target mRNAs. Protein Cell.

[78] Fukao A., Mishima Y., Takizawa N., Oka S., Imataka H., Pelletier J., Sonenberg N., Thoma C., Fujiwara T. (2014). MicroRNAs trigger dissociation of eIF4AI and eIF4AII from target mRNAs in humans. Mol. Cell.

[79] Duncan K.E., Strein C., Hentze M.W. (2009). The SXL-UNR corepressor complex uses a PABP-mediated mechanism to inhibit ribosome recruitment to msl-2 mRNA. Mol. Cell.

[80] Bordeleau M.E., Robert F., Gerard B., Lindqvist L., Chen S.M., Wendel H.G., Brem B., Greger H., Lowe S.W., Porco J.A. (2008). Therapeutic suppression of translation initiation modulates chemosensitivity in a mouse lymphoma model. J. Clin. Investig..

[81] Cencic R., Carrier M., Galicia-Vazquez G., Bordeleau M.E., Sukarieh R., Bourdeau A., Brem B., Teodoro J.G., Greger H., Tremblay M.L. (2009). Antitumor activity and mechanism of action of the cyclopenta[b]benzofuran, silvestrol. PLoS ONE.

[82] Malina A., Cencic R., Pelletier J. (2011). Targeting translation dependence in cancer. Oncotarget.

[83] Fukaya T., Iwakawa H.O., Tomari Y. (2014). MicroRNAs block assembly of eIF4F translation initiation complex in Drosophila. Mol. Cell.

[84] Khaleghpour K., Svitkin Y.V., Craig A.W., DeMaria C.T., Deo R.C., Burley S.K., Sonenberg N. (2001). Translational repression by a novel partner of human poly(A) binding protein, Paip2. Mol. Cell.

[85] Khaleghpour K., Kahvejian A., De Crescenzo G., Roy G., Svitkin Y.V., Imataka H., O’Connor-McCourt M., Sonenberg N. (2001). Dual interactions of the translational repressor Paip2 with poly(A) binding protein. Mol. Cell. Biol..

[86] Sakakibara S., Okano H. (1997). Expression of neural RNA-binding proteins in the postnatal CNS: Implications of their roles in neuronal and glial cell development. J. Neurosci..

[87] Sakakibara S., Nakamura Y., Satoh H., Okano H. (2001). *RNA*-binding protein Musashi2: Developmentally regulated expression in neural precursor cells and subpopulations of neurons in mammalian CNS. J. Neurosci..

[88] Yano M., Hayakawa-Yano Y., Okano H. (2016). RNA regulation went wrong in neurodevelopmental disorders: The example of Msi/Elavl RNA binding proteins. Int. J. Dev. Neurosci. Off. J. Int. Soc. Dev. Neurosci..

[89] Kawahara H., Imai T., Imataka H., Tsujimoto M., Matsumoto K., Okano H. (2008). Neural RNA-binding protein Musashi1 inhibits translation initiation by competing with eIF4G for PABP. J. Cell Biol..

[90] Cragle C., MacNicol A.M. (2014). Musashi protein-directed translational activation of target mRNAs is mediated by the poly(A) polymerase, germ line development defective-2. J. Biol. Chem..

[91] Moretti F., Kaiser C., Zdanowicz-Specht A., Hentze M.W. (2012). PABP and the poly(A) tail augment microRNA repression by facilitated miRISC binding. Nat. Struct. Mol. Biol..

[92] Yi H., Park J., Ha M., Lim J., Chang H., Kim V.N. (2018). PABP Cooperates with the CCR4-NOT Complex to Promote mRNA Deadenylation and Block Precocious Decay. Mol. Cell.

[93] Haghighat A., Mader S., Pause A., Sonenberg N. (1995). Repression of cap-dependent translation by 4E-binding protein 1: Competition with p220 for binding to eukaryotic initiation factor-4E. EMBO J..

[94] Gingras A.C., Kennedy S.G., O’Leary M.A., Sonenberg N., Hay N. (1998). 4E-BP1, a repressor of mRNA translation, is phosphorylated and inactivated by the Akt(PKB) signaling pathway. Genes Dev..

[95] Gingras A.C., Gygi S.P., Raught B., Polakiewicz R.D., Abraham R.T., Hoekstra M.F., Aebersold R., Sonenberg N. (1999). Regulation of 4E-BP1 phosphorylation: A novel two-step mechanism. Genes Dev..

[96] Yang H.S., Jansen A.P., Komar A.A., Zheng X., Merrick W.C., Costes S., Lockett S.J., Sonenberg N., Colburn N.H. (2003). The transformation suppressor Pdcd4 is a novel eukaryotic translation initiation factor 4A binding protein that inhibits translation. Mol. Cell. Biol..

[97] Palamarchuk A., Efanov A., Maximov V., Aqeilan R.I., Croce C.M., Pekarsky Y. (2005). Akt phosphorylates and regulates Pdcd4 tumor suppressor protein. Cancer Res..

[98] Liwak U., Thakor N., Jordan L.E., Roy R., Lewis S.M., Pardo O.E., Seckl M., Holcik M. (2012). Tumor suppressor PDCD4 represses internal ribosome entry site-mediated translation of antiapoptotic proteins and is regulated by S6 kinase 2. Mol. Cell. Biol..

[99] Sarkissian M., Mendez R., Richter J.D. (2004). Progesterone and insulin stimulation of CPEB-dependent polyadenylation is regulated by Aurora A and glycogen synthase kinase-3. Genes Dev..

[100] Mendez R., Hake L.E., Andresson T., Littlepage L.E., Ruderman J.V., Richter J.D. (2000). Phosphorylation of CPE binding factor by Eg2 regulates translation of c-mos mRNA. Nature.

[101] Atkins C.M., Nozaki N., Shigeri Y., Soderling T.R. (2004). Cytoplasmic polyadenylation element binding protein-dependent protein synthesis is regulated by calcium/calmodulin-dependent protein kinase II. J. Neurosci..

[102] Huang Y.S., Jung M.Y., Sarkissian M., Richter J.D. (2002). N-methyl-D-aspartate receptor signaling results in Aurora kinase-catalyzed CPEB phosphorylation and alpha CaMKII mRNA polyadenylation at synapses. EMBO J..

[103] Schmidlin M., Lu M., Leuenberger S.A., Stoecklin G., Mallaun M., Gross B., Gherzi R., Hess D., Hemmings B.A., Moroni C. (2004). The ARE-dependent mRNA-destabilizing activity of BRF1 is regulated by protein kinase B. EMBO J..

[104] Benjamin D., Schmidlin M., Min L., Gross B., Moroni C. (2006). BRF1 protein turnover and mRNA decay activity are regulated by protein kinase B at the same phosphorylation sites. Mol. Cell. Biol..

[105] Mobarak C.D., Anderson K.D., Morin M., Beckel-Mitchener A., Rogers S.L., Furneaux H., King P., Perrone-Bizzozero N.I. (2000). The RNA-binding protein HuD is required for GAP-43 mRNA stability, GAP-43 gene expression, and PKC-dependent neurite outgrowth in PC12 cells. Mol. Biol. Cell.

[106] Lim C.S., Alkon D.L. (2012). Protein kinase C stimulates HuD-mediated mRNA stability and protein expression of neurotrophic factors and enhances dendritic maturation of hippocampal neurons in culture. Hippocampus.

[107] Fujiwara T., Fukao A., Sasano Y., Matsuzaki H., Kikkawa U., Imataka H., Inoue K., Endo S., Sonenberg N., Thoma C. (2012). Functional and direct interaction between the RNA binding protein HuD and active Akt1. Nucleic Acids Res..

[108] van Gorp A.G., van der Vos K.E., Brenkman A.B., Bremer A., van den Broek N., Zwartkruis F., Hershey J.W., Burgering B.M., Calkhoven C.F., Coffer P.J. (2009). AGC kinases regulate phosphorylation and activation of eukaryotic translation initiation factor 4B. Oncogene.

[109] Raught B., Peiretti F., Gingras A.C., Livingstone M., Shahbazian D., Mayeur G.L., Polakiewicz R.D., Sonenberg N., Hershey J.W. (2004). Phosphorylation of eucaryotic translation initiation factor 4B Ser422 is modulated by S6 kinases. EMBO J..

[110] Shahbazian D., Roux P.P., Mieulet V., Cohen M.S., Raught B., Taunton J., Hershey J.W., Blenis J., Pende M., Sonenberg N. (2006). The mTOR/PI3K and MAPK pathways converge on eIF4B to control its phosphorylation and activity. EMBO J..

[111] Rogers G.W., Richter N.J., Lima W.F., Merrick W.C. (2001). Modulation of the helicase activity of eIF4A by eIF4B, eIF4H, and eIF4F. J. Biol. Chem..

[112] Eom T., Muslimov I.A., Tsokas P., Berardi V., Zhong J., Sacktor T.C., Tiedge H. (2014). Neuronal BC RNAs cooperate with eIF4B to mediate activity-dependent translational control. J. Cell Biol..

[113] Chi S.W., Zang J.B., Mele A., Darnell R.B. (2009). Argonaute HITS-CLIP decodes microRNA-mRNA interaction maps. Nature.

[114] Hafner M., Landthaler M., Burger L., Khorshid M., Hausser J., Berninger P., Rothballer A., Ascano M., Jungkamp A.C., Munschauer M. (2010). Transcriptome-wide identification of RNA-binding protein and microRNA target sites by PAR-CLIP. Cell.

[115] McGlincy N.J., Ingolia N.T. (2017). Transcriptome-wide measurement of translation by ribosome profiling. Methods.

[116] Ingolia N.T., Hussmann J.A., Weissman J.S. (2019). Ribosome Profiling: Global Views of Translation. Cold Spring Harb. Perspect. Biol..

